# Effect of different levels of dietary vitamin E on reproductive and productive performances in Japanese quails *(Coturnix coturnix japonica)*

**Published:** 2017-12-15

**Authors:** Parvin Abedi, Saleh Tabatabaei Vakili, Morteza Mamouei, Ali Aghaei

**Affiliations:** *Department of Animal Science, Faculty of Animal Science and Food Technology, Ramin Agriculture and Natural Resource University of Khuzestan, Mollasani, Ahvaz, Iran*

**Keywords:** Egg quality, Japanese quail, Reproduction, Vitamin E

## Abstract

The aim of this study was to evaluate the effects of dietary vitamin E on reproductive and productive parameters in Japanese quails. A total number of 240 female and 80 male Japanese quail were divided into five treatments with four replications in a completely randomized design. Experimental diets were zero control, 30, 60, 120 and 240 mg kg^-1^ of vitamin E. Fertility and total hatchability were not affected by treatments. But, the lowest hatch of fertile eggs and the highest embryonic death were observed in control group (*p* < 0.05). Left testes weight in T2 and T4 was higher than control (*p* < 0.05). Right testes weight and sera FSH concentration in males were not affected by treatments. The highest testosterone concentration of males was observed in T5 (*p* < 0.05). Weight and length of oviduct as well as weight of ovary and FSH concentration in females did not affected by treatments. Estrogen concentration in T4 treatment was greater than control (*p* < 0.05). Most of the egg characteristics were not affected by treatments. However, higher egg mass and production rates were observed in T2 and T3groups than control group (*p* < 0.05). Feed intake and conversion ratio did not differ among treatments. In conclusion, dietary vitamin E improved hatch of fertile egg, embryonic viability, egg mass and production rates in Japanese quail. The effect of dietary vitamin E combined with selenium on these characteristics are recommended for future study in Japanese quail.

## Introduction

Vitamin E is one of the active natural antioxidants that is used in animal feeding. It exhibits an antioxidant activity at low concentration and pro-oxidant activity at high concentration.^1^ Vitamin E is primarily known as an antioxidant in reducing cellular free radical damage.^2^ Dietary vitamin E was found to improve reproduction and antioxidant capability of breeder chickens.^3^^,^^4^ It was speculated that vitamin E protects the liver from lipid peroxidation and prevents cell membranes from damage.^5^ Antioxidants play an important role in avian reproduction. 

In the chicken, vitamin E prevents lipid peroxidation of spermatozoa and enhances semen quality and fertilizing ability.^6^ The association of vitamin E deficiency with impaired male reproduction has been established for three decades, and traditionally it is called the ‘anti-sterility’ vitamin.^7^ In addition to its role in reproduction, vitamin E is the major fat-soluble antioxidant, which breaks the chain reaction of lipid peroxidation. The nutrients required for chicken embryo development are derived from the nutrients stored in the eggs, and vitamin E can be increased by raising the dietary level of this vitamin. Thus, the incorporation of vitamin E into the egg may theoretically both increase oxidative stability and provide a source of tocopherols for human nutrition and health.^8^ In broiler diets, supplementing with vitamin E at levels higher than NRC^9^ requirement had better live weight gain and feed conversion ratio.^10^ Animals are unable to synthesize tocopherol and are dependent solely on dietary sources. Therefore, dietary supplementation of vitamin E gives positive effects on production and reproduction traits of animals as well as poultry species.^11^

Vitamin E supplementations have been used for enhancing productive and reproductive performances of chicken and semen characteristic of male Japanese quail.^7^^,^^12^ This vitamin has a positive effect on growth performance of poultry by increasing resistance to disease and stress.^6^ Inadequate doses of Vitamin E in the basal diet is resulted with slow growth rate.^13^ The objective of this study was to determine the possible beneficial effects of dietary vitamin E supplementation on reproductive and productive performances in Japanese quails. 

## Materials and Methods

**Location and experimental design.** The present research was performed on a research farm of Ramin Agriculture and Natural Resources University of Khuzestan in Iran (latitude 31°52′N and longitude 48°53′E). For this purpose, a total number of 320 adult Japanese quails were used. Upon arrival the birds into the research center (35 weeks old), they were keep two weeks for acclimation and then were trained for 42 days in cage system under temperature and humidity conditions of 21 ˚C and 60%, respectively. This research was done in a completely randomized design with five treatments, four replicates and 16 observations (12 female and four male Japanese quails in each replicate). The basal diet ingredients and constituents are presented in Table 1. Treatments were supplemented diets with increasing levels of zero or control, 30, 60, 120 and 240 mg kg^-1^ of vitamin E Premix (Aras E-Vimix^®^, Pharmaceutical Co., Amol, Iran). Each kg contains 5500 IU of vitamin E or α-tocopherol acetate. Birds received the diet and water *ad libitum*. The lighting regime was 16 hr light and 8 hr of darkness per day.

**Table 1:** Ingredients and constituents of Japanese quails base diet.

**Ingredients **	**Percentage**
**Maize**	56.15
**Soybean meal**	30
**Fish meal**	3
**Soybean oil**	2.95
**Dicalcium phosphate**	1
**Sodium chloride**	0.30
**Oyster shell**	5.80
**Methionine**	0.25
**Lysine**	0.05
**Vitamin premix** ^1^	0.25
**Mineral premix** ^2^	0.25
***Constituents ***	
**ME(Kcal kg** ^-1^ **)**	2900
**Crude Protein **	20.04
**Calcium **	2.50
**Phosphorus available **	0.40
**Lysine **	1.14
**Methionine **	0.59
**Methionine+ Cysteine **	0.90

1 Vitamin premix contained (per kilogram of diet): vitamin A, 8,800 IU; vitamin D3, 2,000 IU; vitamin E, 11 IU; vitamin K3, 2.20 mg; vitamin B12, 0.015 mg; vitamin B1, 1.40 mg; vitamin B2, 4 mg; vitamin B6, 3 mg; folic acid, 1 mg; choline, 1,000 mg; nicotinic acid, 30 mg and pantothenic acid, 10 mg.

2 Mineral premix contained (per kilogram of diet): manganese, 99.20 mg; zinc, 84.70 mg; iron, 50 mg; copper, 10 mg; iodine, 1 mg; and selenium, 0.20 mg.

**Productive parameters.** The daily egg production and egg weights were recorded for six weeks in each replicate. Every two weeks, five eggs from each replicate were selected randomly and quantity and quality traits of egg like shape index, shell (percent, thickness, breaking strength), albumen (percent, weight, pH, diameter and height), yolk (percent, weight, pH, diameter and height) and egg mass were estimated as described by Singh and Panda.^14^ Haugh unit score of egg was calculated by the method which was described by Kondaiah *et al.*^15^ Feed consumption was recorded weekly by calculating the average daily feed consumption of the hens in each replicate. Egg weight was measured using all eggs produced during two consecutive days every week. Egg mass was calculated as laying rate × egg weight, whereas feed conversion (feed consumption / egg mass) was calculated every week throughout the experimental period.

**Reproductive parameters.** At the end of experiment (day 42), 40 eggs from each replicate were incubated under standard condition (37.70 ˚C and 55% humidity of setter conditions for 15 days and 37.20 ˚C and 65% of hatcher conditions for 3 days; automatic rotation every 1 hr; in a poultry egg incubator) and reproductive traits included the fertility rate (number of fertile eggs / total numbers eggs placed into incubator)×100, hatchability of incubated eggs (number of hatched chicks / total number of egg placed into incubator) × 100, hatchability of fertile eggs (number of hatched chicks / number of fertile eggs placed into incubator) × 100 and embryonic mortality percent were evaluated.^16^ At day 42, two birds (one male and one female) from each replicate were selected randomly and slaughtered for evaluation the reproductive system morphometric and gonadal parameters as well as values of blood serum reproductive hormones (testosterone and FSH in males and estrogen and FSH values in females). Estrogen and testosterone concentrations were determined by ELISA kit (Monobind Inc., CostaMesa, USA). The value of FSH was evaluated by ELISA kit (Pishtaz-Teb Co., Tehran, Iran).

**Statistical analysis. **Statistical analysis was performed using the SPSS (version 16.00; SPSS Inc., Chicago, USA). Differences in mean percentages of the variables were analyzed by one-way repeated analysis of variance. Significance between means at the 5% was tested using Duncan’s multiple comparison test (post-hoc). The percentages were reported as mean ± SE. 

## Results

The effect of dietary levels of vitamin E on fertility, hatchability and embryonic mortality of Japanese quails is presented in Table 2. The fertility and hatch of incubated eggs (total hatchability) were not affected by treatments (*p* > 0.05). While, the hatch of fertile eggs was increased in all vitamin E groups when compared to control group (*p* < 0.05). Embryonic mortality rate in treatments supplemented with vitamin E at levels of 30, 60 and 120 mg kg^-1^ was significantly lower than control (without vitamin E supplemented). In control group, the embryonic mortality rates of hatcher and early incubation periods were 94.29% and 5.71%, respectively. In treatments supplemented with vitamin E at levels of 30, 60 and 120 mg kg^-1^, total of embryonic mortalities were recorded in hatcher period. In T5 group, 69.90% of embryonic death was observed in hatcher period and remain in early incubation period. 

The effect of dietary vitamin E supplementation on testis weights and blood serum testosterone and FSH levels is shown in Table 3. Left testis weight in diets supplemented with 30 and 120 mg kg^-1^ vitamin E was higher than control and other treatments (*p* < 0.05). Right testis weight was not affected by treatments. The highest sera testosterone concentration in male birds was recorded in 240 mg kg^-1^ vitamin E (*p* < 0.05). There was no significant difference of blood serum FSH concentration in male Japanese quails (*p* > 0.05). 

The effect of supplemented vitamin E in diet on reproductive performance of female Japanese quails is shown in Table 3. The oviduct weight and length as well as weight of ovary were not affected by treatments (*p* > 0.05). F1, F2 and F4 follicular weight were not influenced by treatments. The lowest F3 follicular weight was observed in 60 mg kg^-1^ of vitamin E. There was no difference in F1-F4 follicular diameter among treatments (*p* > 0.05). Blood serum estrogen level of female Japanese quails in 120 mg kg^-1^ vitamin E was higher than control, 30 and 240 mg kg^-1^ vitamin E (*p* < 0.05). In females, there were no differences in concentration of FSH between treatments (*p* > 0.05). 

The effect of dietary vitamin E levels on egg characteristics of Japanese quails is presented in Table 4. Except for egg mass and egg production percent, other egg characteristics were not affected by treatments (*p* > 0.05). Egg mass and egg production percent were significantly higher in 30 and 60 mg kg^-1 ^of vitamin E treatments than control (*p* < 0.05). As shown in Table 4, feed intake and feed conversion ratio were not affected by treatments (*p* > 0.05).

**Table 2:** Effect of different levels of vitamin E (mg kg^-1^) in diet on fertility, hatchability and embryonic death in Japanese quails.

**Parameters**	**T 1**	**T 2**	**T 3** **‎**	**T 4** **‎**	**T 5** **‎**
**Fertility (%)**	87.85 ± 1.37	89.28 ± 2.95	84.29 ± 4.44	80.67 ± 0.79	86.67 ± 1.78
**Hatch of incubated eggs (** **%** **)**	76.31 ± 8.90	87.86 ± 2.15	81.91 ± 2.93	78.37 ± 0.49	76.42 ± 4.10
**Hatch of fertile egg (** **%** **) **	85.42 ± 2.20^b^	96.0 ± 2.35^a^	96.22 ± 2.19^a^	95.03 ± 1.77^a^	91.64 ± 1.48^a^
**Embryonic mortality (** **%** **)**	12.59 ± 2.92^a^	3.57 ± 2.14^b^	4.81 ± 1.65^b^	4.48 ± 1.50^b^	7.14 ± 0.83^ab^

Means ± SE within a row without a common superscript differ significantly (*p* < 0.05). T1, T2, T3, T4 and T5 represent the birds in the treatments supplemented with vitamin E at levels of zero (control), 30, 60, 120 and 240 mg kg^-1^, respectively.

**Table 3:** Effect of different levels of vitamin E (mg kg^-1^) in diet on male and female reproductive characteristics in Japanese quails.

**Parameters**	**T 1**	**T 2**	**T 3** **‎**	**T 4** **‎**	**T 5** **‎**
**Left testis weight (g)**	2.77 ± 0.08^b^	3.83 ± 0.21^a^	3.05 ± 0.13^b^	3.88 ± 0.35^a^	2.82 ± 0.23^b^
**Right testis weight (g)**	2.70 ± 0.11	3.37 ± 0.22	3.08 ± 0.09	3.37 ± 0.36	2.73 ± 0.27
**Oviduct weight (g)**	8.53 ± 0.52	7.78 ± 1.16	8.17 ± 0.94	7.60 ± 0.75	7.50 ± 0.38
**Oviduct length (cm)**	34.50 ± 1.74	35.13 ± 1.33	34.50 ± 0.87	35.13 ± 1.33	31.50 ± 0.29
**Ovary weight (g)**	6.40 ± 0.90	5.60 ± 1.05	4.75 ± 0.39	6.85 ± 1.28	6.55 ± 1.16
**F1 follicular weight (g)**	2.12 ± 0.49	2.93 ± 0.26	2.3 ± 0.15	2.95 ± 0.46	3.00 ± 0.56
**F2 follicular weight (g)**	1.58 ± 0.33	1.50 ± 0.38	1.05 ± 0.12	1.87 ± 0.38	1.77 ± 0.31
**F3 follicular weight (g)**	0.60 ± 0.07^b^	1.05 ± 0.06^a^	0.48 ± 0.06^c^	1.16 ± 0.02^a^	0.78 ± 0.15^ab^
**F4 follicular weight (g)**	0.46 ± 0.12	0.68 ± 0.18	0.40 ± 0.06	0.52 ± 0.14	0.42 ± 0.15
**F1 follicular diameter (cm)**	1.67 ± 0.09	1.88 ± 0.09	1.64 ± 0.04	1.82 ± 0.14	1.84 ± 0.09
**F2 follicular diameter (cm)**	1.44 ± 0.15	1.40 ± 0.13	1.20 ± 0.07	1.23 ± 0.24	1.43 ± 0.07
**F3 follicular diameter (cm)**	0.98 ± 0.10	1.02 ± 0.15	0.78 ±0.04	1.08 ± 0.12	1.02 ± 0.12
**F4 follicular diameter (cm)**	0.67 ± 0.10	0.88 ± 0.15	0.58 ± 0.04	0.80 ± 0.14	0.69 ± 0.07
**Female estrogen (pg mL** ^-1^ **)**	472.00 ± 178.53^bc^	328 ± 30.74^c^	841.38 ± 1.79^ab^	1000.98 ± 13.04^a^	322 ± 85.89^c^
**Female FSH (IU mL** ^-1^ **)**	0.28 ± 0.1	0.16 ± 0.08	0.10 ± 0.03	0.22 ± 0.09	0.11 ± 0.04
**Male testosterone (ng mL** ^-1^ **)**	6.23 ± 1.49^b^	5.30 ± 1.11^b^	5.48 ± 1.44^b^	6.00 ± 1.10^b^	11.35 ± 0.62^a^
**Male FSH (IU mL** ^-1^ **)**	0.20 ± 0.04	0.33 ± 0.10	0.38 ± 0.16	0.34 ± 0.22	0.18 ± 0.03

Means ± SE within a row without a common superscript differ significantly (*p* < 0.05). T1, T2, T3, T4 and T5 represent the birds in the treatments supplemented with vitamin E at levels of zero (control), 30, 60, 120 and 240 mg kg^-1^, respectively.

**Table 4:** Effect of different levels of vitamin E in diet on egg characteristics and feed performance in Japanese quails.

**Parameters**	**T 1**	**T 2**	**T 3** **‎**	**T 4** **‎**	**T 5** **‎**
**Egg production (%)**	73.11 ± 2.64^c^	81.26 ± 2.95^ab^	86.70 ± 1.72^a^	77.32 ± 2.94^bc^	74.90 ± 0.91^bc^
**Shape index**	80.89 ± 1.15	76.98 ± 1.32	78.93 ± 1.70	77.43 ± 1.35	78.01 ± 0.80
**Haugh units**	88.40 ± 1.94	87.22 ± 2.45	88.38 ± 1.08	90.34 ± 1.33	88.23 ± 1.15
**Shell (%)**	15.70 ± 0.93	16.11 ± 0.62	14.38 ± 0.76	14.18 ± 0.30	15.36 ± 0.49
**Yolk (%)**	34.84 ± 0.75	33.94 ± 0.30	34.16 ± 0.94	35.35 ± 0.67	35.18 ± 0.80
**Albumen (%)**	49.47 ± 1.14	49.95 ± 0.86	51.47 ± 1.05	50.47 ± 0.67	49.85 ± 0.70
**Egg weight ** **)** **g** **(**	13.61 ± 0.52	13.68 ± 0.32	13.61 ± 0.37	13.57 ± 0.38	13.89 ± 0.37
**Egg shell thickness (mm)**	0.24 ± 0.04	0.21 ± 0.01	0.20 ± 0.01	0.20 ± 0.01	0.24 ± 0.04
**Shell breaking strength** ** (kg cm** ^-2^ **)**	0.24 ± 0.04	0.21 ± 0.01	0.20 ± 0.01	0.20 ± 0.01	0.22 ± 0.01
**Albumen weight (g)**	6.30 ± 0.35	6.46 ± 0.14	7.00 ± 0.27	6.63 ± 0.23	6.77 ± 0.33
**Yolk weight (g)**	4.43 ± 0.22	4.14 ± 0.14	4.19 ± 0.17	4.10 ± 0.11	4.28 ± 0.22
**Albumen pH**	8.02 ± 0.27	8.31 ± 0.10	8.20 ± 0.26	7.86 ± 0.22	8.03 ± 0.16
**Yolk pH**	7.16 ± 0.19	7.18 ± 0.19	7.16 ± 0.18	7.65 ± 0.21	7.14 ± 0.19
**Albumen diameter (cm)**	5.06 ± 0.15	4.88 ± 0.04	5.32 ± 0.25	4.96 ± 0.10	4.97 ± 0.13
**Yolk diameter (cm)**	2.80 ± 0.10	2.70 ± 0.04	2.74 ± 0.07	2.80 ± 0.04	2.78 ± 0.05
**Albumen height (mm)**	4.60 ± 0.36	4.43 ± 0.39	4.56 ± 0.20	4.92 ± 0.21	4.57 ± 0.21
**Yolk height (mm)**	11.54 ± 0.37	11.77 ± 0.35	12.20 ± 0.35	12.11 ± 0.30	12.57 ± 0.29
**Egg mass (g per day)**	8.87 ± 0.30^b^	10.44 ± 0.41^a^	10.55 ± 0.35^a^	9.56 ± 0.45^ab^	8.97 ± 0.18^b^
**Feed intake (g per day)**	24.11 ± 0.78	24.83 ± 0.41	25.16 ± 0.78	24.02 ± 1.43	21.16 ± 0.78
**Feed conversion ratio**	2.72 ± 0.05	2.40 ± 0.11	2.40 ± 0.09	2.52 ± 0.15	2.36 ± 0.10

Means ± SE within a row without a common superscript differ significantly (*p* < 0.05). T1, T2, T3, T4 and T5 represent the birds in the treatments supplemented with vitamin E at levels of zero (control), 30, 60, 120 and 240 mg kg^-1^, respectively.

## Discussion

Vitamin E is essential for optimum fertility and hatchability.^17^ In agreement with our observation, Hargis suggest that supplementation of vitamin E on broiler breeder chicken diet reduced embryonic mortality.^18^ However, Lin *et al.* reported that diet supplemented with vitamin E did not significantly influence embryonic survival rate in Taiwan native chickens.^3^ The research with different avian species has shown that increased level of dietary vitamin E increased hatchability and fertility.^3^^,^^19^ In contrast, Hooda *et al.* found that feeding higher rates of dietary vitamin E did not affect the fertility and hatchability in quails.^20^ Amiri-Andi *et al.* reported the positive effect of vitamin E on fertility and hatchability in chicken.^21^ In our findings, fertility and total hatchability traits of quails were not influenced by dietary vitamin E supplementation. However, hatch of fertile egg rate was increased in all of the dietary vitamin E levels when compared to control group. Vitamin E can be transferred from the diet to the egg and consequently to the developing embryo.^22^ Surai and Fisinin reported that increased vitamin E and selenium supplementation of maternal diet is beneficial for embryonic and early postnatal development of chicks.^23^ The transfer of these antioxidants to the offspring reduces hatching stress. Survivability could be attributed to reserves of the antioxidants acquired from the maternal diet and accumulated during embryo-genesis which helped to boost post hatch survival, thereby increasing the survival chance of the birds.^24^ Results from this study showed that, supplementing Japanese quail diet with vitamin E at 30, 60 and 120 mg kg^-1^ improved embryonic survivability. Lin *et al.* reported that fertility rate was decreased when basal diets were provided but increased when vitamin E was added into chicken diet.^3^ Vitamin E deficiency adversely affects the fertility of Japanese quail.^7^ No significant differences were observed with fertility and hatchability in the present study. This was consistent with reports that dietary vitamin E did not improve the hatchability rate in chickens.^3^ Fertility and hatchability were not affected by 25, 50, 75 and 100 mg kg^-1^ vitamin E supplements in broiler breeders.^25^ In cockerels, dietary supplementation of vitamin E in 20 to 160 mg kg^-1^ had no effect on fertility.^4^ Also, feeding of higher rates of vitamin E did not affect the fertility and hatchability of male and female Japanese quails.^20^ However, Muduuli *et al.* observed that different levels of dietary supplemented vitamin E improved the hatchability rate of chickens.^26^ Vitamin E has been reported to be essential for normal hatchability;^27^ whereas another study^28^ and also our study (for total hatchability but no for hatch of fertile eggs) have not found vitamin E supplementation to improve hatchability. In a study, the 1^st^ week of vitamin E depletion, embryonic mortality was increased approximately similar to the 1^st^ and 3^rd^ week of development, however, thereafter the mortality was dominant during the 1st week.^29^ On the other hand, Leeson *et al.* found no clear-cut trends in embryonic mortality distribution or in types of malformations and malpositions.^30^ Lin *et al.* reported an improvement of 7.00 and 13.00% in fertility and hatchability of total eggs set, respectively, when Taiwan native pullets were given 80 mg kg^-1^ of supplemental vitamin E, as compared with control group, however, there was no difference in hatchability of fertile eggs set and embryo survival.^3^ Some symptoms in the vitamin E-deficient embryo include cloudy spots in the eyes, blindness, abnormal vascular system (resulting in exudative diathesis and hemorrhages), and stunting.^31^ Other factors, such as selenium, anti-oxidants, and types of fats, interact with vitamin E and complicate the understanding of its role in embryonic development.^32^ Dietary supplementation of vitamin E at 0, 20, 40, 80 and 160 mg kg^-1^, did not significantly affect plasma testosterone concentration in Taiwan native roosters,^4^ that was similar to our findings in vitamin E levels lower than 240 mg kg^-1^. However, sera testosterone concentration was increased in 240 mg kg^-1^ of vitamin E group in present research. Surai reported increased testes weight when ganders were fed with 20 to 40 IU Vitamin E.^12^ However, plasma testosterone concentration has been positively correlated with body weight.^33^ In one study, adult male quails receiving moderate supplemental vitamin E (75 and 150 IU kg^-1^) had a higher testicular weight and plasma testosterone than quails fed on either vitamin E deficient or more highly supplemented diets with 225 and 300 IU α-toco-pherol acetate per kg.^20^ In our study, low and moderate levels of vitamin E (30 and 60 mg kg^-1^) caused the higher egg production rate and egg mass than control group. However, other egg quality traits were not affected by vitamin E supplementation. Similarly, Bollengier-Lee *et al.* and Ciftci *et al.* showed that vitamin E supplementation significantly increased egg production in laying hens exposed to heat stress.^34^^,^^35^ However, Puthpongsiriporn *et al. *found that the addition of vitamin E (25, 45 or 65 IU per kg) to hen diets decreased the detrimental effects of heat stress in laying hens, and it increased the egg quality.^36^ Additionally, Cherian *et al.* reported that the increase in yolk vitamin E concentration and the incorporation of tocopherols in hen diets prevents lipid oxidative deterioration.^37^ Dietary supplementation with vitamin E improves egg production by facilitating the release of vitellogenin from the liver and by increasing its concentration into blood circulation.^34^ In agreement with present study, previous researches showed that egg weight, feed intake, and feed conversion were not significantly affected by vitamin E in laying hens during heat stress.^38^ However, the feed intake rate in laying hens exposed to a chronic heat stress was not affected by 125 mg of α-tocopheryl acetate per kg in diet.^36^ Our results showed that yolk color, eggshell breaking strength, eggshell thickness, and Haugh units were not significantly influenced by dietary vitamin E levels. These results were in agreement with Irandoust *et al.*, who found that supplementation with 250 mg kg^-1^ of vitamin E had no significant effect on egg quality.^39^ In contrast, the hens fed diets containing vitamin E had an increased yolk percentage and a lower albumen percentage when compared to control hens.^35^ Supplementation with 200 mg kg^-1^ of vitamin E significantly improved egg production and yolk percentage in laying hens.^40^ With evaluation the effect of vitamin E on production performance and egg quality traits in Indian native Kadaknath hen, production performance in terms of body weight, egg weight and hatchability did not differ significantly, whereas egg production and fertility rates were significantly higher in 150 IU vitamin E per kg compared to the control (15 IU vitamin E per kg) and 300 IU vitamin E per kg. Egg quality traits in terms of albumin weight, yolk weight, shell thickness, albumin index and yolk index did not differ significantly, whereas the Haugh unit score was significantly higher in 150 IU vitamin E per kg than the control and 300 IU vitamin E per kg.^41^ In another study, supplementation of 85 IU vitamin E increased feed conversion, Haugh unit and egg production in laying hens exposed to heat stress.^42^

In evaluation the vitamin E effect on reproductive and productive performances in turkey, feed intake showed no significant variation between control (the basal diet) and 250 mg kg^-1^ vitamin E supplementation treatments. However, hatchability was significantly higher in combination of 125 mg kg^-1^ vitamin E and 0.15 mg kg^-1^ selenium when compared to those on control, 250 mg kg^-1^ vitamin E and 0.30 mg kg^-1^ selenium supplementation. Also, no significant differences were observed for average hatched weight, feed intake and feed conversion ratio of the pullets. Survivability was increased significantly by the supplemented treatments than control. Birds on combination of 125 mg kg^-1^ vitamin E and 0.15 mg kg^-1^ selenium had significantly higher value of average survival weight than those on control.^43^

It was concluded that some reproductive and productive traits of Japanese quails could be affected by different levels vitamin E in diet. In conclusion, dietary vitamin E improved some of reproductive and productive traits such as hatch of fertile egg, embryonic viability, egg mass and production rates. Sera estrogen concentration of female quails in 120 mg kg^-1^ of vitamin E was higher than control. The highest sera testosterone level of male quails was recorded in 240 mg kg^-1^ of vitamin E. The use of vitamin E in combination with selenium because of their synergistic effect is recommended for future study in Japanese quail. 
